# Optimizing water and nitrogen management in a wheat–maize rotation system: synergistic increases in grain yield, resource use efficiency, and economic and environmental benefits

**DOI:** 10.3389/fpls.2026.1769742

**Published:** 2026-02-11

**Authors:** Weilin Kong, Chunhua Gao, Fengtao Zhao, Feiyan Ju, Zongxin Li, Haijun Zhao, Kaichang Liu, Ping Liu

**Affiliations:** 1 State Key Laboratory of Nutrient Use and Management, Key Laboratory of Agro-Environment in Huang-Huai-Hai Plain, Institute of Agricultural Resources and Environment, Shandong Academy of Agricultural Sciences, Jinan, China; 2 Institute of Industrial Crops, Shandong Academy of Agricultural Sciences, Jinan, China; 3 Shandong Academy of Agricultural Sciences, Jinan, China

**Keywords:** controlled-release fertilizer, drip irrigation, economic and carbon benefits, water–nitrogen coupling, wheat–maize rotation

## Abstract

**Introduction:**

Optimizing water and nitrogen management is crucial for the sustainable development of wheat–maize rotation system. This study systematically examined the impacts of various water and nitrogen management strategies on the wheat–maize rotation system, with the aim of identifying integrated practices that can simultaneously improve yield, resource use efficiency, economic returns, and environmental outcomes.

**Methods:**

A field experiment was conducted from 2022 to 2024 in Tai’an, Shandong Province, China. Strategies involved different nitrogen fertilizers (compound fertilizer, urea, and controlled-release fertilizer) and irrigation methods (flood, drip irrigation DI, and micro-sprinkler irrigation SI). Outcomes were assessed based on yield, water and nitrogen use efficiency, economic benefits, and environmental performance, using entropy-weighted TOPSIS comprehensive evaluation. This research aiming to identify an optimized management practice that can simultaneously. enhance both economic and carbon benefits(carbon sequestration/emission ratio).

**Results and discussion:**

Drip irrigation with 60% basal controlled-release fertilizer and 40% top-dressed urea (T4) yielded optimal results. Compared to conventional flood irrigation with 50% basal compound fertilizer and 50% top-dressed urea, T4 synergistically increased annual system yield by 1.08–3.99%, improved water use efficiency to 9.42 kg·m^-3^ and nitrogen use efficiency to 34.75%, achieved the highest net income (24,347.9 CNY·ha^-1^), and raised carbon benefits to 21.38. Entropy-weighted TOPSIS comprehensive evaluation further demonstrated that the T4 treatment under drip irrigation obtained the highest closeness coefficient (0.702). These findings show that integrating drip irrigation with the split application of controlled-release fertilizer and urea can facilitate the efficient alignment of water and nitrogen resources. This approach is a viable technical pathway for promoting sustainable and low-carbon production under the wheat–maize rotation system in the Huang–Huai–Hai region of China.

## 1 Introduction

Global food security and the sustainable development of agriculture are significant challenges at present. Therefore, optimizing the agricultural system as a whole, rather than focusing on single factors, is crucial. This requires a systems approach that can reveal the synergistic interactions between core components like water and nitrogen, and evaluate potential trade-offs and synergies between productivity, efficiency, and environmental goals (Michalczyk et al., 2014; Yang et al., 2024). Water scarcity and the excessive use of nitrogen fertilizer are major barriers that hinder the green transformation of agriculture in many regions (Wang N. et al., 2023; Zhang et al., 2022), but particularly in the Huang–Huai–Hai region of China where the dominant wheat–maize rotation system has historically been characterized by low water use efficiency (WUE) and nitrogen use efficiency (NUE) (Zhang et al., 2025; Ju et al., 2009), and significant environmental pollution risks (Chen et al., 2025). The ineffective management of water and nitrogen resources results in waste and elevated production costs, as well as leading to a range of environmental problems, including soil degradation, nitrate accumulation in groundwater, and increased greenhouse gas emissions (Li et al., 2024; Zhang et al., 2021).

Recent studies have focused on the development of innovative nitrogen fertilizer types and application methods with the aim of enhancing the NUE while minimizing losses. Guo et al. (2024) demonstrated that the application of controlled-release urea significantly reduced the volatilization of ammonia, increased N_2_O emissions, and contributed to higher yields in summer maize. Similarly, Hou et al. (2024) reported that the split application of urea, tailored to the crop nitrogen demand, effectively enhanced both the winter wheat yield and NUE. Furthermore, smart irrigation and precise water management have emerged as prominent research areas. Integrating drip irrigation with soil moisture sensors has been shown to significantly increase the yield and WUE in wheat and maize (Yang M. D. et al., 2020; Zain et al., 2023). In addition, micro-sprinkler irrigation was shown to be effective at enhancing the micro-environment of the crop canopy. Studies indicate that this method optimizes the soil water distribution and reduces losses through evaporation to improve the WUE. In wheat–maize rotation systems, the integrated application of micro-sprinkler and drip irrigation can achieve water-saving outcomes comparable to those of drip irrigation, while simultaneously enhancing the crop growth environment and promoting healthier plant development (Zhang et al., 2024).

Recent studies have highlighted the importance of “water–nitrogen coupling” as a central strategy for optimizing resource allocation. In addition to plant genetics, studies indicate that appropriately managing water and nitrogen resources is crucial for reducing the yield gap in major cereal crops (Sinclair and Rufty, 2012). Sufficient soil moisture is crucial for the dissolution of nitrogen, and its transport and absorption by plant roots. Optimal nitrogen nutrition facilitates root growth and canopy photosynthesis, thereby improving the WUE of crops (Su et al., 2022). The synergistic interaction between water and nitrogen supplies ultimately optimizes crop growth and development, increasing yields and enhancing the resource use efficiency. From a physiological perspective, this synergy is driven by a bidirectional feedback loop. Water availability fundamentally regulates nitrogen acquisition and metabolism: adequate soil moisture facilitates the mass flow and diffusion of mineral nitrogen (NO_3_^-^ and NH_4_^+^) to root surfaces, thereby determining uptake rates. Conversely, nitrogen nutrition critically shapes plant water relations and use efficiency. Adequate nitrogen promotes deeper root proliferation, enhancing access to soil water reserves. It also elevates photosynthetic capacity and leaf area development by supplying essential components for chlorophyll and photosynthetic proteins, thereby increasing biomass production per unit of water transpired—i.e., intrinsic water use efficiency. Thus, effective water–nitrogen coupling coordinates these reciprocal processes, creating a positive feedback loop that maximizes the efficiency of both resources.

Several studies have demonstrated that combining drip irrigation systems with strategic nitrogen management can further improve the WUE and NUE to increase economic benefits and enhance environmental sustainability (Gupta et al., 2023; Zheng et al., 2020). In addition, Li et al. (2020) demonstrated that applying fertigation through drip irrigation has the potential to reduce greenhouse gas emissions and enhance soil organic carbon levels. Despite these documented benefits, the combined adoption of drip irrigation and controlled-release fertilizers in smallholder wheat-maize systems of the North China Plain remains limited, primarily due to higher initial investment costs and a lack of tailored management recommendations. Moreover, previous research mainly focused on individual crop growth cycles or isolated seasons, thereby limiting our understanding of the dynamic coupling mechanisms between water and nitrogen, and their alignment with crop requirements on an annual scale. Similarly, there have been few comprehensive assessments of different water–nitrogen management strategies, particularly from the perspectives of economic benefits and environmental sustainability, and multi-dimensional benefit comparisons across different combinations of irrigation methods and nitrogen fertilizer types are lacking. Therefore, the present study, through a two-year field experiment in a conventional wheat–maize rotation system, systematically tested combinations of different irrigation methods and nitrogen fertilizer types/strategies, and applied the entropy-weighted TOPSIS method for a comprehensive evaluation, aiming to fill these knowledge gaps.

Based on previous research, the present study examined a conventional wheat–maize rotation system in a two-year field experiment. The experiment systematically tested combinations of different nitrogen fertilizer types, i.e., compound fertilizer, urea, and controlled-release fertilizer, and different irrigation methods, i.e., flood irrigation, drip irrigation, and micro-sprinkler irrigation. We hypothesized that the integration of drip irrigation with a split application of controlled-release and conventional urea would optimize water-nitrogen coupling, leading to simultaneous improvements in yield, resource use efficiency, economic returns, and carbon benefits in a wheat-maize rotation system. The objectives of this study were: (1) to assess the effects of different water–nitrogen management strategies on the annual crop yield and its components; (2) to assess the effects of different water–nitrogen management strategies on the annual WUE and NUE; (3) to quantify the differences in economic benefits and environmental carbon emissions among treatments, and assess the multi-dimensional benefits; and (4) to apply the entropy-weighted TOPSIS method for a comprehensive evaluation to identify the optimal management strategy that maximizes system-level efficiency and synergies across agronomic, economic, and environmental dimensions of the agricultural system. The results obtained in this study should provide a theoretical foundation and technical basis for achieving efficient, low-carbon, and sustainable grain production.

## 2 Materials and methods

### 2.1 Experimental site

**Figure 1:**
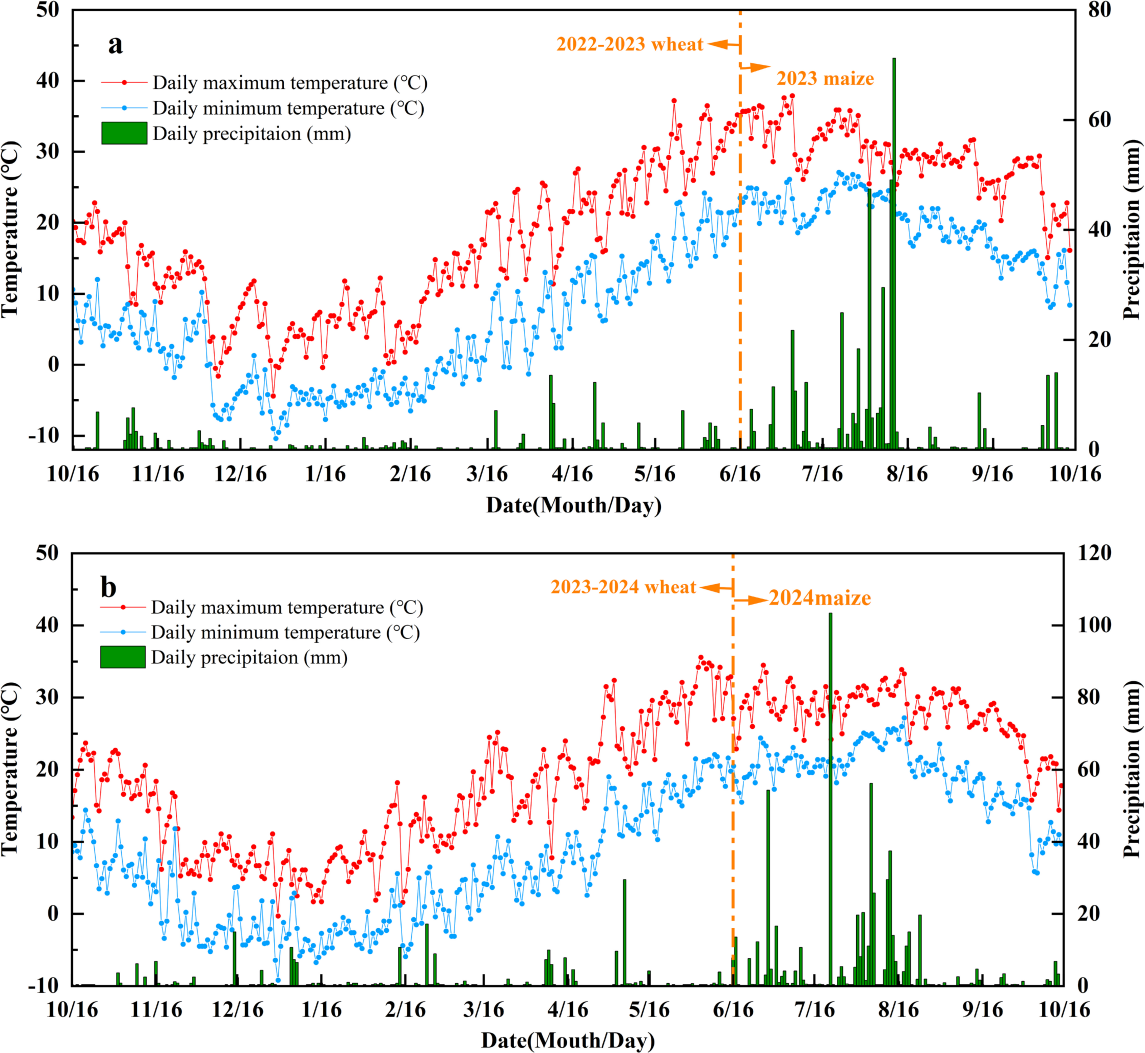
Daily minimum and maximum air temperatures, and rainfall amounts at the experimental site during 16 October 2022 to 16 October 20233 **(A)** and 16 October 2023 to 16 October 2024 **(B)**.

The experiment was conducted between 2022 and 2024 at the Experimental Farm located in Tai’an City, Shandong Province, China (36°0′N, 117°0′E). The study site is characterized by a temperate continental semi-humid monsoon climate, with an average annual temperature of 12.8°C and average annual precipitation of 700 mm. A winter wheat–summer maize double cropping system is implemented annually at this site. Winter wheat is typically sown in mid-October and harvested in early June in the subsequent year, followed by sowing maize in mid-to-late June and harvesting in early October in the same year. All crop residues are chopped and reincorporated into the field. **Figure 1** illustrates the precipitation amounts and daily average temperatures recorded at the experimental site during the winter wheat and summer maize growing seasons from 2022 to 2024. The soil at the site is classified as cinnamon soil with a clay texture and flat topography. The initial physicochemical properties of the topsoil (0–20 cm soil depth) were as follows: pH = 7.14, organic matter content = 20.02 g kg^–1^, total nitrogen = 1.21 g kg^–1^, total phosphorus = 1.66 g kg^–1^, and total potassium = 15.12 g kg^–1^.

### 2.2 Experimental design

This experimental study evaluated two crop varieties comprising winter wheat (Taikemai 33) and maize (Denghai 605), and the following three different types of nitrogen fertilizer: compound fertilizer (N-P_2_O_5_-K_2_O: 15-15-15) commonly utilized by local farmers, urea (46% N), and controlled-release urea (CRU Kingenta Ecological Engineering Group Co.,Ltd.) combined with normal urea (CRUNU) fertilizer. CRU had a polymer coating designed for a release duration of approximately 90 days. Three irrigation techniques were implemented: flood irrigation, which is prevalent among local farmers, drip irrigation, and micro-sprinkler irrigation. The experiment employed a split-plot arrangement with irrigation method as the main plot factor. However, the sub-plot (fertilization) treatments were not fully factorial across all main plots. Flood irrigation (conventional practice) included only two nitrogen management treatments (CK1 and T1), whereas drip and micro-sprinkler irrigation (optimized practices) each included nine treatments (CK2 and T1-T7). This design allowed for a focused comparison of optimized systems against the conventional baseline. The area of each plot was 200 m^2^ (10 m × 20 m), and plots were separated by a 1-m buffer zone to prevent interference by nitrogen and irrigation between adjacent treatments.

The fertilizer application rates were as follows: conventional farming practice using 250 kg N ha^–1^, 112 kg P_2_O_5_ ha^–1^, and 112 kg K_2_O ha^–1^; and an optimized input fertilization practice using a reduced total input rate (195 kg N ha^–1^, 97.5 kg P_2_O_5_ ha^–1^, and 97.5 kg K_2_O ha^-^¹), which served as the common baseline for testing different nitrogen fertilizer types and split strategies under water-saving irrigation. For each nitrogen treatment, basal fertilizers were used before the sowing stage, while topdressing was applied during the jointing stage (wheat) and Tasseling stage (maize). All treatments, including the zero-nitrogen controls (CK1 under flood irrigation; CK2 under drip and micro-sprinkler irrigation), received the same basal applications of phosphorus and potassium fertilizers as described for the ‘optimized fertilization rate. The application rates were applied consistently to both winter wheat and summer maize in succession on the same plots. Phosphorus and potassium fertilizers were administered as basal applications prior to sowing. Nitrogen fertilizers were distributed manually for the specific fertilizer types and application rates described in the experimental protocol for each treatment. Wheat seeds in the first year were sown on 16 October, 2022 and harvested on 10 June, 2023, and the second year commenced on October 17, 2023 and completed on 7 June, 2024. Maize seeds in the first year were sown on 12 June, 2023 and harvested on 15 October, 2023, and the second year commenced on June 10, 2024 and completed on 15 October, 2024.

The irrigation volumes varied slightly between years due to factors such as climate, irrigation methodology, and crop type. Irrigation was applied four times during the winter wheat growing season. The total water amounts applied by conventional flood irrigation were 2775 m^3^ ha^–1^ in the 2022–2023 season and 1575 m^3^ ha^–1^ in the 2023–2024 season. By contrast, under the optimized drip irrigation treatment, 1950 m^3^ ha^–1^ was applied in 2022–2023 and 1080 m^3^ ha^–1^ in 2023–2024. Irrigation was applied three times in the summer maize growing season. The conventional flood irrigation amounts were 1575 m^3^ ha^–1^ in 2023 and 1275 m^3^ ha^–1^ in 2024, and optimized drip irrigation involved the application of 1125 m^3^ ha^–1^ in 2023 and 870 m^3^ ha^–1^ in 2024. Due to increased evaporation rates, the total water applied under optimized micro-sprinkler irrigation exceeded that by drip irrigation. The drip lines were installed with a spacing of 45 cm between lines and emitters were positioned every 30 cm, delivering a flow rate of 2 L h^–1^. Micro-sprinkler lines were arranged with a spacing of 1.8 m. Irrigation scheduling was primarily based on crop growth stages to ensure water supply during critical periods (e.g., jointing, booting, filling for wheat; bell stage and grain filling for maize). The specific volumes for ‘optimized’ drip and micro-sprinkler treatments were designed to achieve approximately 60-70% of the irrigation amount used in conventional flood irrigation (F), reflecting a water-saving target while maintaining adequate soil moisture. The exact volumes were adjusted between years in response to seasonal precipitation patterns (**Figure 1**) to avoid excessive irrigation and potential waterlogging.

Details of the nitrogen fertilizer and irrigation strategies are provided in **Tables 1** and **Supplementary Table S1**.

**Table 1:** Fertilization and irrigation strategies (CRUNU: controlled-release urea combined with normal urea).

Treatment	Nitrogen fertilizer rates(kg ha^-1^)	Fertilization pattern	Irrigation pattern
CK1	0	local farming practice	Flood
CK2	0	optimized fertilization	Drip irrigation/sprinkling
T1	50% NPK (basal application) + 50% urea (topdressing)	local farming practice	Flood
T2	50% NPK (basal application) + 50% urea (topdressing)	optimized fertilization	Drip irrigation/sprinkling
T3	100% CRUNU (basal application)	optimized fertilization	Drip irrigation/sprinkling
T4	60% CRUNU (basal application) + 40% urea (topdressing)	optimized fertilization	Drip irrigation/sprinkling
T5	40% CRUNU (basal application) + 60% urea (topdressing)	optimized fertilization	Drip irrigation/sprinkling
T6	60% CRUNU (basal application) + 40% urea (basal application)	optimized fertilization	Drip irrigation/sprinkling
T7	40% CRUNU (basal application) + 60% urea (basal application)	optimized fertilization	Drip irrigation/sprinkling

CK2 is a no-nitrogen control under both optimized irrigation systems (drip and micro-sprinkler irrigation).

### 2.3 Data collection

#### 2.3.1 Yield and dry matter

At maturity, representative aboveground biomass were sampled from 30 plants, rinsed with deionized water, dried, wiped, and subsequently oven dried at 75°C until constant weight. The dry matter mass was then recorded. These samples were ground and stored for subsequent nitrogen content analysis. At the same time, a random area of wheat from each experimental plot measuring 10 m^2^ was harvested to assess the yield. The grain moisture content was measured using a grain moisture meter (PM-8188-A, KETT, Japan), and the actual yield was standardized to the national grain storage moisture content of 13%. In parallel, yield components were determined from three additional, randomly placed 1m^2^ quadrats per plot. The number of spikes (wheat) or ears (maize) per square meter was counted. Additionally, 30 spikes or ears were randomly selected to determine the average kernel number per spike/ear. The 1000-kernel weight was measured from a bulk grain sample after adjustment to standard moisture content (13% for wheat, 14% for maize).

#### 2.3.2 Soil moisture and nitrogen

Soil samples were collected concurrently with plant harvesting at physiological maturity. Using a five-point sampling method, soil cores were taken from the plow layer (0–20 cm) and subsoil layer (20–40 cm) in each plot. Samples from the same depth were thoroughly mixed to form a composite sample for subsequent analysis of soil moisture and nitrogen content.

The soil moisture content was determined using the time domain reflectometry method. Soil nitrate nitrogen and ammonium nitrogen were analyzed using a flow-injection autoanalyzer (Gu et al., 2015).

#### 2.3.3 Plant nitrogen

The nitrogen content of the aboveground plant parts was determined using the semi-micro Kjeldahl method (Wang et al., 2015).

#### 2.3.4 Resource utilization efficiency assessment

The irrigation WUE was calculated as:


WUE=Y/I

where WUE is the irrigation water use efficiency (kg m^–3^), Y is the crop yield (kg ha^–1^), and I is the irrigation water volume (m3 ha^–1^).

The NUE was calculated as:


$$NUE=\frac{T_{n-}C_{n}}{N}$$

where NUE is the nitrogen use efficiency (%), T_n_ is the nitrogen uptake by aboveground plant parts in the fertilized plot (kg ha^–1^), C_n_ is the nitrogen uptake by aboveground plant parts in the control plot (kg ha^–1^), and N is the nitrogen application rate (kg ha^–1^).

#### 2.3.5 Direct economic benefit assessment

The economic benefits were calculated as:


EB=Y×UP−I−F−S−M−L

where EB is the economic benefit (CNY ha^–1^), Y is the crop yield (kg ha^–1^), UP is the unit price of grain in the month of the harvest that year (CNY kg^–1^), I is the irrigation cost (CNY ha^–1^), F is the fertilizer cost (CNY ha^–1^), S is the seed cost (CNY ha^–1^), M is the machinery cost (CNY ha^–1^), and L is the land rent cost (CNY ha^–1^).

#### 2.3.6 Estimation of crop carbon sequestration

Based on the photosynthesis equation for green plants (6CO_2_ + 6H_2_O → C_6_H_12_O_6_ + 6O_2_) and the dehydration condensation principle for sugars, the production of 162 kg of dry matter requires the absorption and fixation of 264 kg of CO_2_. Thus, for every 1.63 kg of CO_2_ absorbed by the crop, 1.00 kg of dry matter is produced, and 1.00 kg of CO_2_ contains 0.27 kg of carbon. The amount of carbon sequestered was calculated directly from crop dry matter as


$$W_{CO_{2}}=W\times 1.63$$

$$W_{C}=W_{CO_{2}}\times 0.27=W\times 0.44$$

where W_CO2_ represents the amount of CO_2_ fixed per unit area by the crop (kg ha^–1^) and W_C_ represents the amount of carbon sequestered per unit area by the crop (kg ha^–1^). W represents the dry matter content (kg ha^–1^). The coefficient 0.44 is derived from the stoichiometry of photosynthesis (1.63 kg CO_2_ fixed per kg dry matter × 0.27 kg C per kg CO_2_).

#### 2.3.7 Calculation of cultivated land carbon emission differences

Agricultural carbon emissions are mainly due to greenhouse gas emissions associated with anthropogenic farming activities. In the Chinese agricultural context, carbon emissions due to crop cultivation are primarily from the following sources: the use of inputs such as chemical fertilizers, pesticides, and agricultural film; diesel consumption by agricultural machinery; electricity used for irrigation; and organic carbon loss due to tillage practices. Given that the variations between treatments in the present study were mainly due to differences in nitrogen fertilizer application and irrigation practices, the IPCC carbon emission coefficient method was employed to quantify and compare carbon emissions from these two factors across different treatments (Note: This assessment focused on the carbon emissions most directly influenced by water-nitrogen management differences; thus, emissions from pesticides, agricultural film, etc., were not included, defining the boundary of this evaluation), as follows:


$$C=\sum c_{i}=\sum N_{i}\times K_{i}$$

where C represents the total carbon emissions from fertilizer and irrigation for each treatment (kg ha^–1^), C_i_ represents the agricultural carbon emissions from the i-th carbon source, N_i_ represents the input amount of the i-th carbon source, and K_i_ is the carbon emission coefficient for the i-th carbon source. The emission coefficient for chemical fertilizer is 0.8956 kg C kg^–1^ (West and Marland, 2002). The emission coefficient for irrigation electricity was primarily based on the “Provincial Greenhouse Gas Inventory Compilation Guide” issued by the Ministry of Ecology and Environment of the People’s Republic of China: 0.6410 kg C kW^–1^ h^–1^ for the experimental region. The flow rate of the farmland water pump was 40 L h^–1^ and the power was 5.5 kW.

#### 2.3.8 Entropy-weighted TOPSIS evaluation

The entropy-weighted Technique for Order Preference by Similarity to Ideal Solution (TOPSIS) method was selected for the comprehensive evaluation. The entropy-weighted TOPSIS procedure was implemented in two sequential stages. First, objective weights for each evaluation indicator were derived using the entropy method. The initial decision matrix was normalized to render different indicators comparable. The information entropy of each indicator was then calculated; a lower entropy value reflects greater data variability and thus a higher information content contributed by that indicator. Subsequently, the divergence degree and the final objective weight for each indicator were determined. These weights, generated objectively from the dataset, were applied to construct the weighted normalized decision matrix. In the second stage, the classic TOPSIS algorithm was applied to this weighted matrix. The positive ideal solution (PIS) and negative ideal solution (NIS) were identified. The Euclidean distances from each treatment alternative to the PIS (D_i_^+^) and NIS (D_i_^−^) were computed, ultimately yielding the relative closeness coefficient (Ci=D_i_^−^/(D_i_^+^+D_i_^−^) for comprehensive ranking. This method is particularly suited for multi-criteria decision-making problems aimed at ranking a finite set of alternatives (i.e., our treatments) based on their overall performance across multiple, often conflicting, indicators. as follows:

After normalizing the raw data matrix, the cosine method was used to identify the optimal solution (Z^+^) and worst solution (Z^–^) among the evaluation objects. The distances (D_i_^+^ and D_i_^–^) between each evaluation object and the optimal/worst solutions were then calculated to yield the relative closeness (C_i_) of each evaluation object to the optimal solution. The calculation method was described by Du et al. (2020) and is defined as follows.

The optimal solution Z^+^ consists of the maximum value of each column in Z:


$$\begin{array}{l} {\text{Z}}^{+}=(\max\{{\text{Z}}_{11},{\text{Z}}_{21},\ldots ,{\text{Z}}_{\text{n}1}\},\max\{{\text{Z}}_{12},{\text{Z}}_{22},\ldots ,{\text{Z}}_{\text{n}2}\},\ldots ,\\ \max\{{\text{Z}}_{1\text{m}},{\text{Z}}_{2\text{m}},\ldots \ldots {\text{Z}}_{\text{nm}}\})=({\text{Z}}_{1}^{+},{\text{Z}}_{2}^{+},\ldots ,{\text{Z}}_{m}^{+}) \end{array}$$

The worst solution Z^-^ consists of the minimum value of each column in Z:


$$\begin{array}{l} {\text{Z}}^{+}=(\min\{{\text{Z}}_{11},{\text{Z}}_{21},\ldots ,{\text{Z}}_{\text{n}1}\},\min\{{\text{Z}}_{12},{\text{Z}}_{22},\ldots ,{\text{Z}}_{\text{n}2}\},\ldots ,\\ \min\{{\text{Z}}_{1\text{m}},{\text{Z}}_{2\text{m}},\ldots \ldots {\text{Z}}_{\text{nm}}\})=({\text{Z}}_{1}^{-},{\text{Z}}_{2}^{-},\ldots ,{\text{Z}}_{m}^{-}) \end{array}$$

The closeness of each evaluation object to the optimal and worst solutions:


$${\text{D}}_{i}^{+}=\sqrt{\sum_{\text{j}=1}^{\text{m}}{\left({\text{Z}}_{j}^{+}-{\text{Z}}_{\text{ij}}\right)}^{2}}$$

$${\text{D}}_{i}^{-}=\sqrt{\sum_{\text{j}=1}^{\text{m}}{\left({\text{Z}}_{j}^{-}-{\text{Z}}_{\text{ij}}\right)}^{2}}$$

Calculate the relative closeness C^i^ of each evaluation object to the optimal solution:


$${\text{C}}_{\text{i}}=\frac{{\text{D}}_{i}^{-}}{{\text{D}}_{i}^{+}+{\text{D}}_{i}^{-}}$$

Finally, ranking is performed based on the closeness degree C_i_.

### 2.4 Data analysis

Data were analyzed separately for each irrigation method due to the unbalanced treatment structure across methods. For drip and micro-sprinkler irrigation, a one-way analysis of variance (ANOVA) was performed for each growing season to assess the effects of nitrogen management strategies. Within each ANOVA, treatment means were compared using the Least Significant Difference (LSD) test at a significance level of *p* < 0.05. Microsoft Excel 2010 (Microsoft Corp., Redmond, WA, USA) and Origin 2021 (OriginLab, Hampton, MA, USA) software were used for data processing and graph plotting. Data in graphs were presented as means, with the standard error of the mean indicating the deviation. SPSS 22.0 (IBM Corp., Armonk, NY, USA) statistical software was used to detect significant differences and to calculate correlations. The least significant difference test was used to analyze differences with a significance level of 0.05.

## 3 Results

### 3.1 Effects of water and nitrogen management on crop yield

**Figure 2:**
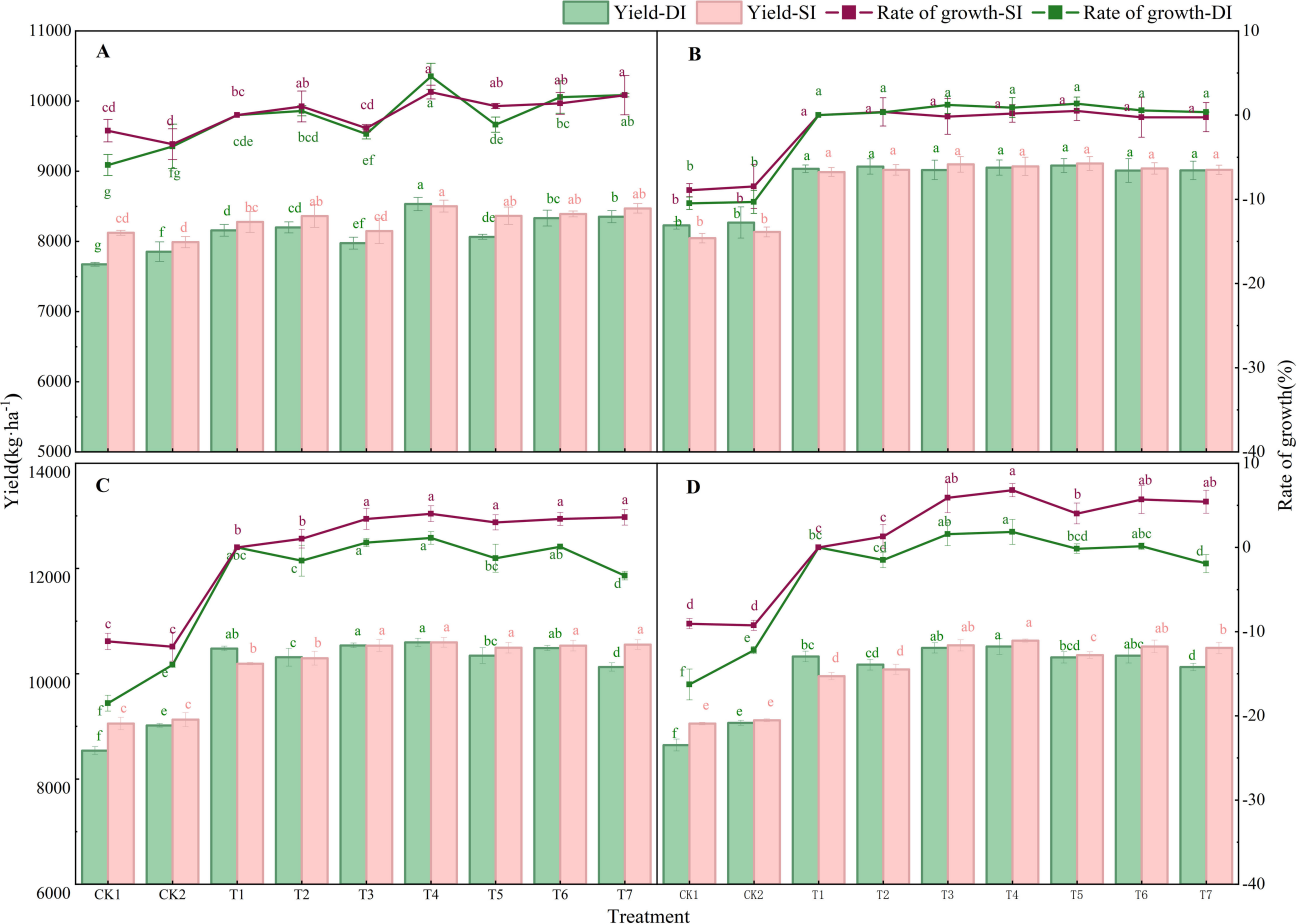
Annual wheat and maize yields under different water and nitrogen management treatments. The main Y-axis represents the wheat and maize yields, and the secondary Y-axis shows the increase in yield compared with T1. **(A)** Wheat yields under drip irrigation (DI) and micro-sprinkler irrigation (SI) during 2022–2023. **(B)** Wheat yields under DI and SI during the period of 2023–2024. **(C)** Maize yields under DI and SI during 2023. **(D)** Maize yields under DI and SI during 2024. In the same sub-figure, bar charts with the same letter indicate no significant difference between treatments (*p* > 0.05), whereas those with different letters denote significant differences between treatments (*p* < 0.05).

The two-year field experiment revealed that water–nitrogen management strategies significantly influenced the annual yield of the wheat–maize rotation system (**Figure 2**). The T4 strategy consistently yielded the highest output under both drip (DI) and micro-sprinkler irrigation (SI).Under drip irrigation, T4 achieved superior yields in both seasons. In 2022–2023, the wheat yield under T4 was 8,532.5 kg ha^-1^, which was 4.6% higher than that under the conventional practice (T1) (*p* < 0.05). The subsequent maize yield in T4 reached 10,596.95 kg ha^-1^, exceeding T1 by 0.75% (*p* < 0.05). This trend continued in 2023–2024, with both wheat and maize yields under T4 being significantly greater than those under T1, CK1, and CK2 (*p* < 0.05). Consequently, the annual system yield under drip irrigation was increased by 2.65% (2022–2023) and 1.08% (2023–2024) relative to T1.Under micro-sprinkler irrigation, T4 also demonstrated significant yield advantages. During 2022–2023, wheat yield under T4 was 8,502.0 kg ha^-1^, representing a 2.73% increase over T1 (*p* < 0.05). Maize yield followed a similar trend. The annual system yield benefits were 3.43% and 3.99% higher than T1 in the 2022–2023 and 2023–2024 seasons, respectively.

Analysis of the correlations between yield components provided further insights into the contributions of various factors to the yield outcomes (**Supplementary Figure S1**). During the wheat growing season (**Supplementary Figure S1A**), a highly significant positive correlation was identified between the yield and both the year and nitrogen application rate (*p* < 0.001), whereas a highly significant negative correlation was found with the irrigation volume (*p* < 0.001). Moreover, the spike number had a highly significant positive correlation with the yield (*p* < 0.001). By contrast, during the maize growing season (**Supplementary Figure S1B**), the yield had highly significant positive correlations with both the nitrogen application rate and method (*p* < 0.001), but no significant correlations with other factors. Thus, the wheat and maize yields were influenced by different factors. Therefore, implementing targeted water–nitrogen management strategies based on the correlations between the yield and various factors is crucial for enhancing annual crop productivity.

### 3.2 Synergistic improvement of water and nitrogen use efficiency

**Figure 3:**
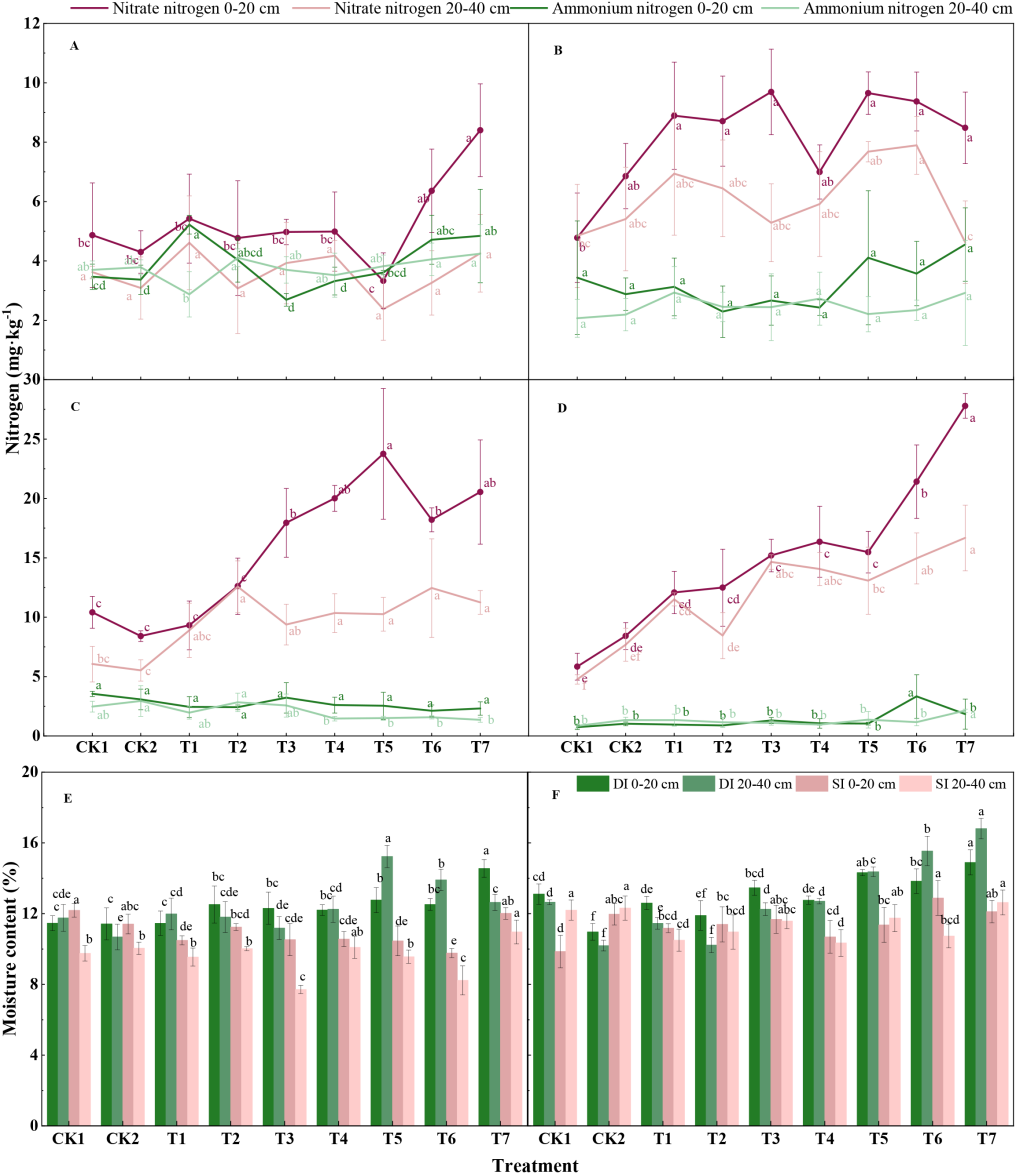
Changes in soil water and nitrogen contents under different water and nitrogen management treatments. **(A, B)** show the nitrate nitrogen and ammonium nitrogen contents in the soil under drip irrigation (DI) **(A)** and micro-sprinkler irrigation (SI) **(B)** at the end of the wheat growing season. **(C, D)** show the nitrate nitrogen and ammonium nitrogen contents in the soil at the end of the maize growing season under DI **(C)** and SI **(D)**. E and F show the soil moisture contents at the end of the experiment in the wheat growing season **(E)** and maize growing season **(F)**. In the same sub-figure, the same letter indicates no significant difference between treatments (*p* > 0.05), whereas different letters denote significant differences between treatments (*p* < 0.05).

**Figure 4:**
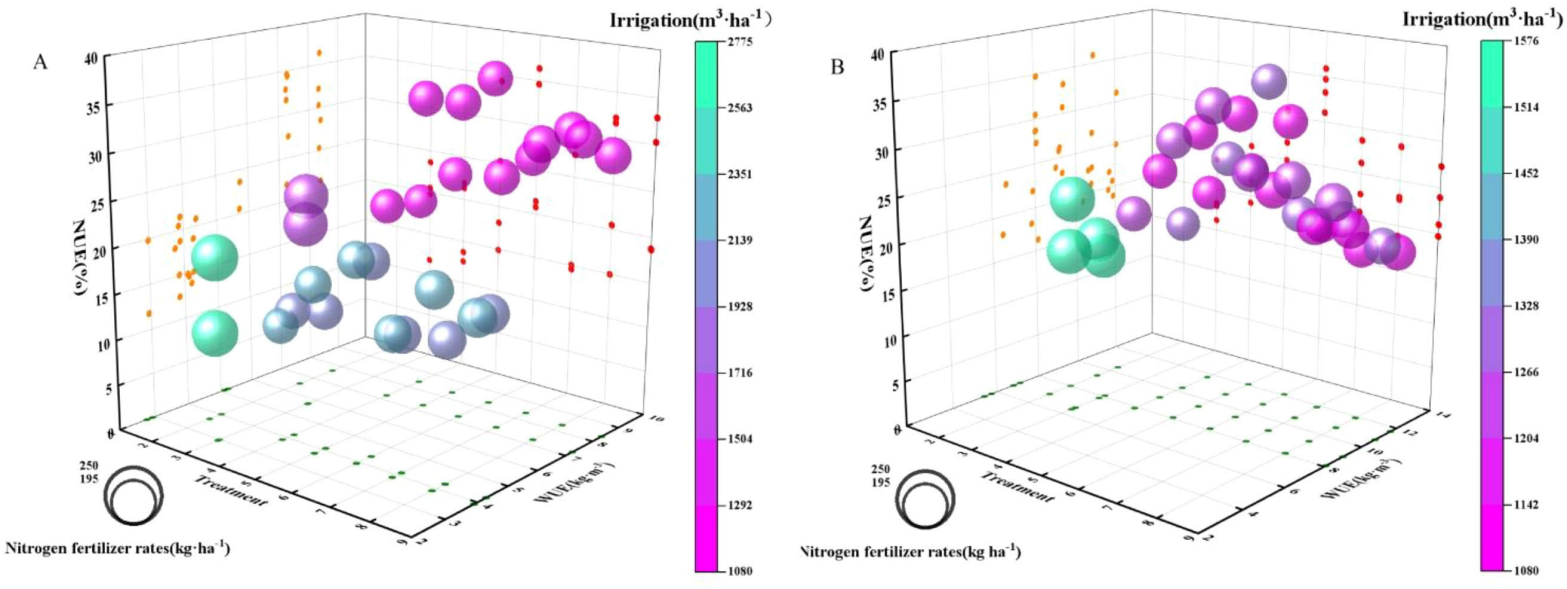
Synergetic relationship between water use efficiency and nitrogen use efficiency. The numbers 1 to 9 on the x-axis correspond to treatments CK1, CK2, T1, T2, T3, T4, T5, T6, and T7, respectively **(A)** Synergistic efficiency of water and nitrogen utilization in wheat. **(B)** Synergistic efficiency of water and nitrogen utilization in maize.

The different water–nitrogen management strategies had significant effects on the soil nitrogen content, as well as on the water status within the wheat–maize rotation system (**Figure 3**). During the wheat growing season (**Figures 3A**, **4B**), the drip irrigation and micro-sprinkler irrigation treatments resulted in significantly higher nitrate-N contents compared with the ammonium-N contents (*p* < 0.05). With drip irrigation, the nitrate-N content in the 0–20 cm soil layer peaked at 8.40 mg kg^–1^ under T7, but this was not significantly different from that under T6. With micro-sprinkler irrigation, the highest nitrate-N content was obtained under T3 at 9.88 mg kg^–1^, but it did not differ significantly from those under T5, T6, and T7. With drip irrigation, the ammonium-N content in the 0–20 cm layer was highest under T1 at 5.22 mg kg^–1^, but not significantly different from those under T6 and T7. With micro-sprinkler irrigation, the ammonium-N content was highest under T7 at 4.24 mg kg^–1^, but not significantly different from those under T2 and T6. In the 20–40 cm layer, the nitrate-N content (ranging from 2.38 to 8.24 mg kg^–1^) was higher than the ammonium-N content (ranging from 2.88 to 4.24 mg kg^–1^). The variations in the nitrate-N contents were greater under micro-sprinkler irrigation (**Figure 3B**) than drip irrigation (**Figure 3A**), suggesting that micro-sprinkler irrigation had a more pronounced effect on changes in the soil nitrate-N distribution during the wheat growing season.

During the maize growing season (**Figures 3C, D**), the soil nitrate-N content was significantly higher under optimized water–nitrogen management compared with conventional farming practices. In particular, with drip irrigation (**Figure 3C**), the nitrate-N concentration in the 0–20 cm soil layer was highest under T5 at 23.76 mg kg^–1^, but it did not differ significantly from those under T4 and T7. By contrast, under micro-sprinkler irrigation (**Figure 3D**), the nitrate-N content in the 0–20 cm soil layer was highest under T7 at 27.80 mg kg^–1^. The ammonium-N contents remained relatively stable across the 0–20 cm and 20–40 cm soil layers, with no significant differences among treatments. Thus, water–nitrogen management during the maize season primarily influenced the activation and accumulation of nitrate-N, but had a smaller impact on ammonium-N levels. At the ends of the wheat (**Figure 3E**) and maize growing seasons (**Figure 3F**), the soil water contents in the 0–20 cm and 20–40 cm soil layers was generally higher under drip irrigation compared with micro-sprinkler irrigation (*p* < 0.05). In particular, during the wheat growing season, drip irrigation increased the water content by 9.21–28.15% in the 0–20 cm layer and by 15.28–69.04% in the 20–40 cm layer. Significant differences in the water contents were found among treatment groups (CK1–T7), indicating that drip irrigation effectively enhanced the soil moisture levels during the maize growing season.

The different water–nitrogen management treatments significantly influenced the synergistic efficiency of water and nitrogen utilization in wheat and maize (**Figure 4**). During the wheat growing season, the synergistic efficiency of water and nitrogen utilization was highest under T4 treatment. WUE and NUE were 4.38 kg m^–3^ and 21.46%, respectively, during 2022–2023, but the values increased to 9.42 kg m^–3^ and 34.75% during 2023–2024 (**Figure 4A**). Thus, under a consistent irrigation volume, optimal nitrogen management could enhance the synergistic effect of water and nitrogen utilization in wheat. Similarly, during the maize growing season, the synergistic efficiency of water and nitrogen utilization was highest under T4 (**Figure 4B**). However, variations in rainfall among crop seasons and years necessitated the application of higher irrigation volumes (1125–2175 m^3^ ha^–1^) during the wheat growing season to achieve high water–nitrogen synergy efficiency. By contrast, the synergistic efficiency of water and nitrogen utilization in the maize growing season was comparable to that in the wheat growing season with relatively lower irrigation volumes (870–1245 m^3^ ha^–1^), indicating the superior efficiency of maize at utilizing external irrigation water. These findings highlight the importance of aligning water and nitrogen inputs in an appropriate manner to achieve high efficiency water and nitrogen utilization.

### 3.3 Economic and environmental effects of wheat-maize rotation system

**Figure 5:**
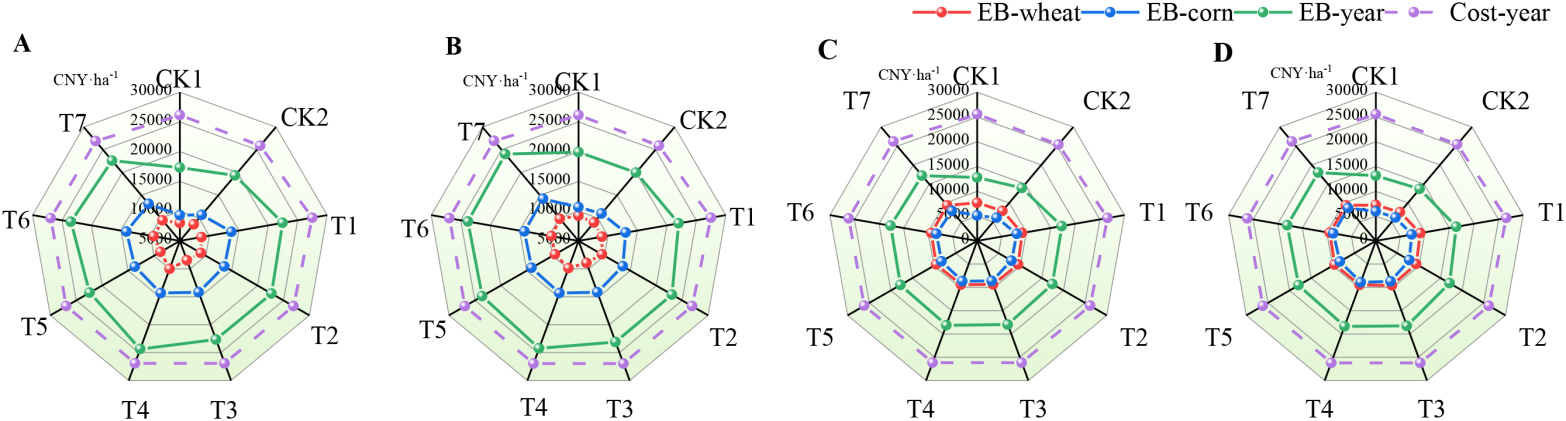
Annual economic performance evaluation results. **(A, B)** show the economic benefit assessment results under drip irrigation (DI) and micro-sprinkler irrigation (SI), respectively, during 2022–2023. **(C, D)** show the economic benefit assessment results under DI and SI, respectively, during 2023–2024.

The different water–nitrogen management strategies, specifically drip irrigation and micro-sprinkler irrigation, and different temporal scales had significant impacts on the annual economic outcomes from the wheat–maize rotation system, as shown in **Figure 5**. During 2022–2023, the highest annual direct economic benefits with both drip irrigation and micro-sprinkler irrigation were obtained under T4 with 24,347.90 CNY ha^–1^ and 24,244.63 CNY ha^–1^, respectively, which were 8.51% and 10.17% higher compared with those under T1 (**Figures 5A, B**). During 2023–2024, the highest economic benefits with both drip irrigation and micro-sprinkler irrigation were obtained under T4 with 18,105.60 CNY ha^–1^ and 18,362.28 CNY ha^–1^, respectively, which were 4.94% and 12.20% higher compared with those under T1 (**Figures 5C, D**). The overall economic returns in 2022–2023 were 26.11% to 51.23% higher than those in 2023–2024 due to variables such as the climatic conditions and fluctuations in agricultural market prices.

**Figure 6:**
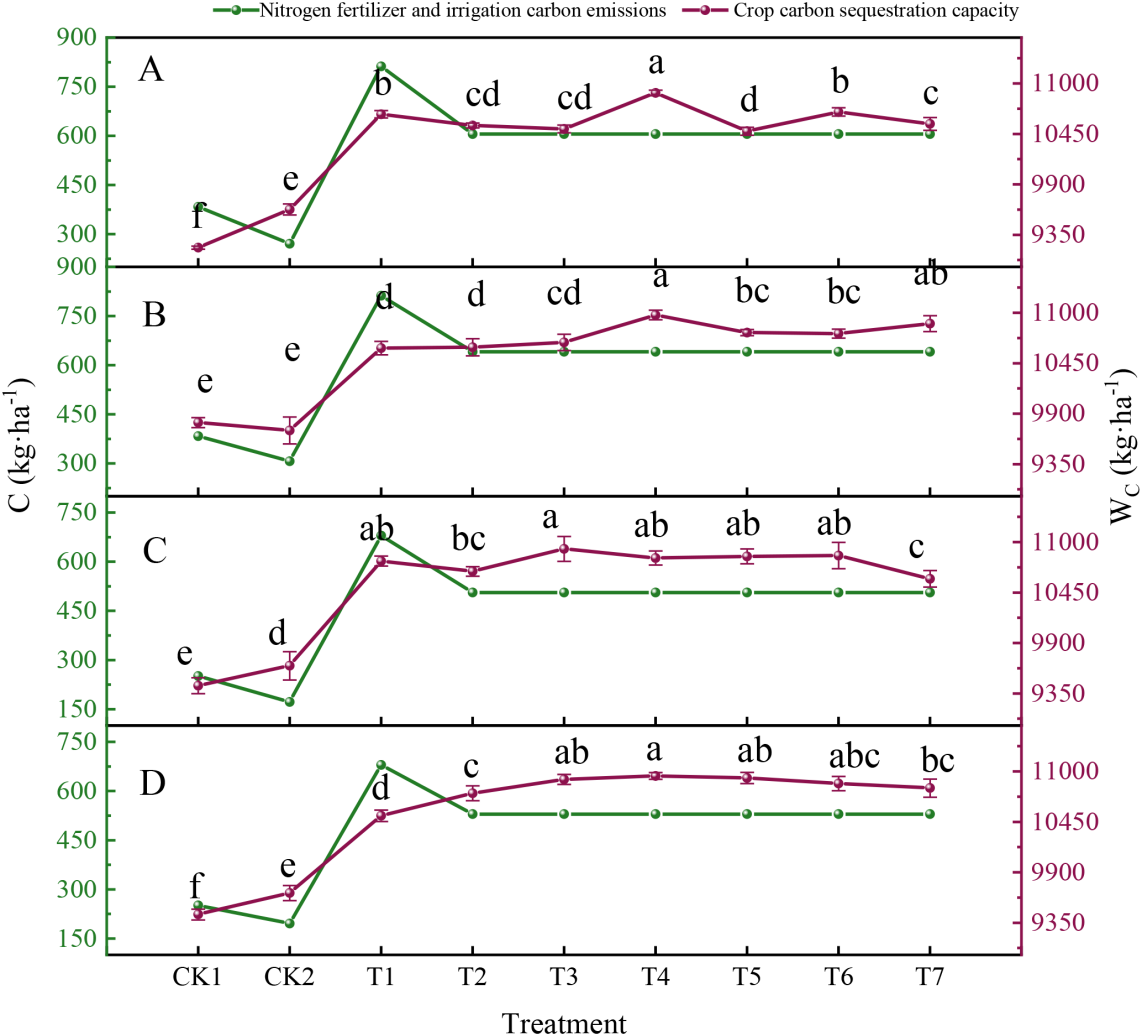
Annual environmental carbon benefits. **(A, B)** show the carbon benefit assessment results under drip irrigation (DI) and micro-sprinkler irrigation (SI), respectively, during 2022–2023. **(C, D)** show the carbon benefit assessment results under DI and SI **(B)**, respectively, during 2023–2024. In the same sub-figure, the same letter indicates no significant difference between treatments (*p* > 0.05), whereas different letters denote significant differences between treatments (*p* < 0.05).

The interactions between water–nitrogen management strategies and temporal scales had great impacts on the synergistic relationship between “nitrogen fertilizer and irrigation carbon emissions” and “crop carbon sequestration capacity” (**Figure 6**). Under T1, the carbon emissions due to nitrogen fertilizer and irrigation increased rapidly to approximately 679 kg C ha^–1^ or higher, before subsequently stabilizing within the range of 506.29 to 641.14 kg C ha^–1^ across optimized treatments T2 through T7. The crop carbon sequestration capacity gradually increased across treatment groups and peaked under T4. The carbon sequestration levels under drip irrigation and micro-sprinkler irrigation were 10,897.21 kg C ha^–1^ and 10,976.56 kg C ha^–1^, respectively, during 2022–2023, and 10,827.05 kg C ha^–1^ and 10,952.15 kg C ha^–1^ during 2023–2024. Thus, the carbon sequestration gains associated with T4 effectively offset carbon emissions. Compared with T1, the highest carbon benefit (ratio of carbon sequestration relative to carbon emissions) was obtained under T4, particularly with drip irrigation, where the carbon benefits were 17.99 in 2022–2023 and 21.38 in 2023–2024, highlighting the significant potential of this method for promoting low-carbon practices.

The economic benefit assessment results (**Figure 5**) showed that the highest net income was obtained under T4 with drip irrigation at 24,347.9 CNY ha^–1^ during 2022–2023, which was 0.43% higher compared with that using micro-sprinkler irrigation in the same period. In terms of environmental benefits (**Figure 6**), the reduced electricity consumption associated with drip irrigation decreased the carbon emissions per unit area by 4.49–5.57% compared with micro-sprinkler irrigation. In particular, the carbon benefits under T4 treatment with drip irrigation exceeded those under other treatment combinations. These findings suggest that drip irrigation can improve economic outcomes as well as having significant potential for promoting low-carbon agricultural production.

### 3.4 Entropy-weighted TOPSIS evaluation of the wheat-maize rotation system

To comprehensively assess the multi-dimensional advantages of various nitrogen management strategies under drip irrigation and micro-sprinkler irrigation, the entropy-weighted TOPSIS method was employed to determine the closeness coefficients to the ideal solution for each treatment during 2022–2023 and 2023–2024 (**Table 2**). The assessment was based on multiple indicators, including the yield, economic benefits, resource utilization efficiency, and environmental benefits. During 2022–2023, the optimal strategy was T4 under both drip irrigation and micro-sprinkler irrigation, with closeness coefficients of 0.702 and 0.694, respectively. The consistency in the closeness coefficients and ranking trends across all treatments suggests that T4 obtained the best synergy among the multi-dimensional “yield–economic–resource–environment” benefits in that year. During the 2023–2024 period, the optimal strategy under both drip irrigation and micro-sprinkler irrigation modes remained T4, with ideal solution closeness values of 0.668 and 0.660, respectively, demonstrating stability across years. Throughout the two experimental years, the closeness coefficients for optimized treatments T2–T7 were generally higher than those for standard farming practice (T1) and conventional controls (CK1 and CK2), highlighting the pivotal role of scientific water–nitrogen regulation in enhancing the comprehensive “yield–economic–resource–environment” benefits and emphasizing the need for optimized annual water–nitrogen management in wheat–maize rotation systems.

**Table 2:** Closeness coefficients to ideal solution for different irrigation and nitrogen fertilizer management strategies.

Treatment	Year	Drip irrigation			Rank	Micro-sprinkler irrigation			
		D^+^	D^-^	Correlation degree		D^+^	D^-^	Correlation degree	Rank
CK1	2022-2023	0.129	0.072	0.358	9	0.119	0.072	0.377	9
CK2		0.105	0.098	0.481	7	0.114	0.09	0.441	7
T1		0.105	0.083	0.44	8	0.103	0.076	0.427	8
T2		0.066	0.102	0.608	4	0.066	0.095	0.590	6
T3		0.069	0.104	0.600	5	0.065	0.102	0.611	5
T4		0.056	0.132	0.702	1	0.056	0.126	0.694	1
T5		0.068	0.1	0.593	6	0.06	0.107	0.641	4
T6		0.063	0.109	0.635	3	0.058	0.113	0.663	3
T7		0.062	0.11	0.641	2	0.057	0.116	0.671	2
CK1	2023-2024	0.121	0.078	0.393	9	0.121	0.082	0.403	8
CK2		0.105	0.099	0.485	7	0.11	0.0987	0.471	7
T1		0.106	0.084	0.442	8	0.109	0.071	0.395	9
T2		0.068	0.101	0.596	6	0.075	0.092	0.550	6
T3		0.065	0.11	0.630	2	0.066	0.115	0.637	2
T4		0.061	0.123	0.668	1	0.064	0.124	0.660	1
T5		0.066	0.107	0.619	3	0.069	0.105	0.604	5
T6		0.066	0.107	0.617	4	0.068	0.109	0.617	3
T7		0.067	0.102	0.603	5	0.069	0.108	0.609	4

The evaluation indicators included: output indicators (wheat yield, maize yield, and annual yield), economic indicators (annual direct economic benefits and annual production material costs), resource utilization efficiency indicators (wheat water use efficiency, maize water use efficiency, wheat nitrogen fertilizer use efficiency, and maize nitrogen fertilizer use efficiency), and environmental benefit indicators (carbon sequestration by crops, and carbon emissions from water and nitrogen treatment).

## 4 Discussion

### 4.1 Impacts of optimized water–nitrogen management on crop yields and associated mechanisms

The findings obtained in this study demonstrate that T4 integrating drip irrigation with a split application of CRUNU and urea produced the highest annual crop yield. In particular, T4 increased the wheat and maize yields by 4.6% and 1.13%, respectively, compared with the conventional farming practice (T1). These results support Ali et al. and Zhang et al. (2020; Zaveri and Lobell, 2019) who reported that the combination of drip irrigation and optimized nitrogen fertilization substantially enhanced crop yields. The observed yield improvements were largely attributed to the precise regulation of root zone moisture facilitated by the drip irrigation system (Gärdenäs et al., 2005), as well as alignment of the nitrogen supply with the crop’s nitrogen demand dynamics through the combined application of CRUNU and urea (Ali et al., 2024; Zhang et al., 2018). Given that the crop yield response is significantly influenced by the availability of water and nutrients within the root zone, the spatial distribution of these resources in the soil is more critical than their surface distribution (Li and Kawano, 1996). The localized and consistent moisture conditions established by drip irrigation directly promote denser root proliferation within the wetted zone to form a more efficient network for water and nutrient uptake, as well as substantially enhancing the mass flow and diffusion of nitrogen toward the root surface, improving its bioavailability. Consequently, the use of drip irrigation is more effective at promoting tillering and ear formation, thereby establishing a robust foundation for developing yield components, as also shown in previous research. Indeed, a highly significant positive correlation was found between the wheat yield and spike number in the present study (*p* < 0.001). Micro-sprinkler irrigation is beneficial for regulating the canopy micro-environment but it distributes water less uniformly than drip irrigation, which may make the nitrogen distribution uneven within the plow layer (Hua et al., 2024), thereby preventing achievement of the full yield potential. We speculate that the less uniform water distribution under micro-sprinkler irrigation (Wang W J. et al., 2023) may lead to uneven nitrogen distribution within the plow layer, potentially increasing the risk of nitrate leaching compared to the more localized application of drip irrigation (Amini et al., 2025; Darwish et al., 2003), which could partly explain the slightly lower yield performance. Furthermore, the analysis of yield components revealed a notable, albeit not always statistically significant, negative correlation between maize yield and irrigation volume across treatments (**Supplementary Figure S1B**). This trend suggests that under the conditions of this study, simply increasing the amount of irrigation water did not guarantee a linear increase in yield, and beyond a certain point, could even be counterproductive. This observation strongly reinforces the central finding of this study: the superiority of the T4 strategy stems not from increased water consumption, but from the optimization of water application (through drip irrigation) coupled with synchronized nitrogen availability. It underscores that achieving higher crop productivity in water-scarce regions hinges on improving water productivity (yield per unit water) through precise management, rather than on maximizing water input. The superiority of the T4 treatment (60% basal CRUNU + 40% top-dressed urea) likely stems from its precise alignment with the dynamic nitrogen demand of the crops. The controlled-release urea provided a stable and extended nitrogen supply, meeting the crop’s demand during the early and middle growth stages, while the top-dressed conventional urea at critical growth stages (e.g., jointing-booting in wheat, bell stage in maize) offered a readily available nitrogen source. This “complementary slow- and fast-release” nitrogen supply pattern, coupled with the stable root-zone moisture environment created by drip irrigation, achieved a high degree of spatiotemporal coupling between water and nitrogen availability. This spatiotemporal coupling mechanism is hypothesized to be a key factor contributing to its superiority over one-time basal application of controlled-release urea (T3) or a high proportion of top-dressed urea (T5).

### 4.2 Enhancement of resource use efficiency through water–nitrogen synergy

In the present study, T4 with drip irrigation obtained the highest water–nitrogen synergy efficiency, with WUE and NUE values of 9.42 kg m^–3^ and 34.75%, respectively, during the wheat growing season. This enhanced efficiency can be explained the following two main mechanisms. First, the drip irrigation system ensures that the soil moisture is stable within the root zone, as shown by the increase in the water content of 9.21–28.15% in the 0–20 cm soil layer compared with micro-sprinkler irrigation, thereby facilitating the improved crop uptake of soil nitrogen, particularly nitrate-N (Singandhupe et al., 2003; Hebbar et al., 2004). In further agreement, the nitrate-N contents were significantly elevated in the 0–20 cm soil layer under T5 and T7 compared with conventional practices during the maize growing season. Second, the slow-release properties of CRUNU fertilizer combined with split urea application effectively aligned with the nitrogen requirements during the critical stages of crop growth, minimizing nitrogen losses during non-essential periods (Qiao et al., 2016; Yang X. Y. et al., 2020).

The synergistic improvement in WUE and NUE under the T4 treatment (**Figure 4**) can be attributed to interconnected physiological processes driven by the specific soil-plant conditions observed. The stable, elevated soil moisture maintained under drip irrigation (**Figure 3**) enhanced nitrogen availability by promoting the mass flow of soil solution and increasing the diffusion of nitrate-N within the root zone. This is supported by the higher soil nitrate-N concentrations measured under optimized treatments, which facilitated greater plant nitrogen uptake—a direct contributor to the enhanced NUE. Concurrently, the optimized nitrogen supply in T4 reciprocally improved the plant’s capacity for water acquisition and use. The sustained nitrogen availability, evidenced by increased aboveground dry matter production, supported the development of a more extensive root system within the moist soil profile and enhanced photosynthetic canopy activity. A robust root system improved water extraction, while adequate nitrogen nutrition elevated photosynthetic carbon gain per unit of water transpired, thereby raising the intrinsic water use efficiency. This reciprocal enhancement—where improved water status boosted nitrogen uptake, and optimized nitrogen supply increased the efficiency of water utilization—constitutes a positive feedback loop. This process-level synergy underlies the system-level gains in both resource use efficiencies documented in this study.

Similarly, Cao et al. (2025) concluded that “water–nitrogen coupling enhances nitrogen retention by delaying nitrogen release and promoting microbial immobilization.” Furthermore, the more coordinated distribution of water and nitrogen in the soil profile under drip irrigation (Haynes, 1990; Hajrasuliha et al., 1998; Rajput and Patel, 2006) may be an important explanation for the superior WUE and NUE synergy under drip irrigation compared with micro-sprinkler irrigation. Therefore, the integration of drip irrigation with the split application of controlled-release and conventional urea essentially optimizes the “spatiotemporal coupling of water and nitrogen in the root zone.” The localized wetted zone created by drip irrigation confines the spatial movement of nitrogen, while the staged release (from controlled-release fertilizer) and supplementation (from top-dressing) of nitrogen synchronize its temporal availability with crop uptake patterns. This synergy minimizes nitrogen losses via leaching and volatilization, thereby significantly enhancing both WUE and NUE, consistent with findings on root-mediated water uptake efficiency (Ober et al., 2021).

### 4.3 System benefits and stability of water–nitrogen management at the annual scale

Water and nitrogen management have critical effects on ecosystem productivity. Previous research has demonstrated that water availability is a key determinant of interannual variability in global terrestrial ecosystem productivity, but nitrogen availability can modulate the effects of climate warming on ecosystem productivity to some degree (Jung et al., 2017). On a global scale, water availability is intricately linked with ecosystem productivity, and nitrogen availability influences the impact of climate warming on productivity by affecting soil nitrogen levels (Liu et al., 2022). In the present field study, despite variations in climate and rainfall between both years, the combination of drip irrigation and optimized nitrogen application (T4 and T6) resulted in remarkably stable yields, resource utilization efficiency, and economic benefits. In particular, the economic returns were highest under T4 during 2022–2023 and 2023–2024, with carbon benefits (in terms of carbon sequestration/emission) of 17.99 and 21.38 under drip irrigation, respectively. This stability suggests that optimized water–nitrogen management can buffer the impacts of interannual climate fluctuations on system productivity to some extent, which is consistent with the study by Chi et al. (2025) who showed that optimized water–nitrogen management strategies can increase crop yields and also enhance the adaptability of crops to climate change. This resilience suggests that optimized water–nitrogen management enhances the robustness of the agricultural system, effectively buffering the impacts of interannual climate fluctuations, a phenomenon also observed in maize under optimized N management (Bonelli and Andrade, 2020). Achieving such stability requires managing for synergistic interactions between resources across the entire annual cycle, rather than optimizing for a single season.

On an annual scale, the water and nitrogen requirements of the wheat–maize rotation system exhibit temporal variability. According to the present study, applying basal CRUNU fertilizer combined with urea topdressing was more suitable for meeting these dynamic demands, thereby facilitating efficient utilization of resources over an annual scale. The optimized nitrogen management strategy (T4) significantly enhanced the crop yield and mitigated greenhouse gas emissions under limited irrigation conditions. This strategy effectively optimized the distribution of nitrogen between wheat and maize, improving the uptake of nitrogen and utilization efficiency, and allowing efficient resource utilization without incurring additional costs (Du et al., 2024). The combined application of controlled-release fertilizer and uncoated urea greatly enhanced the WUE and NUE, increased yields, and decreased the accumulation of soil nitrate and leaching (Zheng et al., 2020).

### 4.4 Pathway to achieving economic and environmental benefits

Optimized water–nitrogen management strategies have been studied extensively in agricultural production, with the main aim of enhancing crop yields while simultaneously reducing emissions. Effective management of water and nitrogen resources can lead to increased crop yields and significant reductions in nitrogen emissions, thereby promoting sustainable agricultural development (Duan et al., 2019). Several studies have demonstrated that enhancing the NUE can yield both economic and environmental benefits. For instance, Langholtz et al. (2021) showed that a 20% improvement in NUE could decrease the nitrogen demand, boost the net profits of farmers, and also reduce nitrate contamination of water bodies. A key finding of this study is that the T4 strategy achieved a highly desirable synergy between economic and environmental objectives, successfully avoiding the trade-offs that often occur between profitability and sustainability in conventional agricultural systems. This aligns with Rosa-Schleich et al. (2019), who emphasized that reducing input costs while maintaining ecosystem services can enhance both farm profitability and environmental outcomes. This “win-win” outcome was achieved by driving the system towards higher system-level efficiency, where resources are converted into marketable yield and sequestered carbon with minimal waste and emission. In the present study, optimized water–nitrogen management under T4 with drip irrigation obtained the highest net income (24,347.9 CNY ha^–1^) and also resulted in significantly lower carbon emissions per unit area compared with conventional farming practices. The enhanced carbon benefits can mainly be explained as follows. First, optimizing the application of nitrogen and implementing water-saving irrigation techniques decreased the usage of fertilizer and consumption of irrigation energy to directly reduce carbon emissions. Second, the increased crop yield enhanced the system’s carbon sequestration capacity, where T4 achieved carbon sequestration ranging from 10,897 to 10,977 kg C ha^–1^. These findings agree with the conclusions of Yao et al. (2025), who indicated that optimized water and nitrogen management can increase the yield and reduce emissions. These findings highlight the potential of applying precise combined water–nitrogen management for significantly enhancing the environmental sustainability of agricultural ecosystems while also maintaining economic viability.

This study was conducted at a single site over two years. Future research should validate these findings across multiple locations and soil types to assess broader applicability. Long-term monitoring is needed to evaluate effects on soil health and carbon dynamics. Investigating the economic feasibility and farmer adoption barriers would also be valuable for policy formulation.

## 5 Conclusions

The findings obtained in the present study demonstrate that integrated water–nitrogen management strategy T4 combining drip irrigation with the split application of 60% basal CRUNU fertilizer and 40% top dressed urea synergistically enhanced crop yields and the resource utilization efficiency, and obtained environmental benefits in the wheat–maize rotation system on the North China Plain. Specifically, this strategy increased the annual system yield by 1.08–3.99%, improved water use efficiency (WUE) and nitrogen use efficiency (NUE) to 9.42 kg m^-3^ and 34.75%, respectively, achieved the highest net income of 24,347.9 CNY ha^-1^, and raised the carbon benefit (carbon sequestration to emission ratio) to 21.38, compared to conventional practice. This integrated water–nitrogen management strategy improved the synchrony between water availability and nitrogen release timing, as evidenced by the enhanced soil nitrate-N content in the root zone (**Figure 4**) and the significantly higher WUE and NUE. This approach significantly improved annual system-level efficiencies in terms of water and nitrogen use, and provided a favorable rhizospheric environment for resource uptake by maintaining higher and more synchronized available levels of water and nitrogen. Comprehensive evaluations using the entropy-weighted TOPSIS method consistently identified T4 as the optimal model because it obtained the highest benefits in terms of the yields, economic returns, resource utilization, and environmental performance. Thus, the T4 strategy is recommended for large-scale demonstration in the Huang–Huai–Hai region, providing a scientific basis for achieving green development and “Dual Carbon” goals in agriculture.

## Data Availability

The raw data supporting the conclusions of this article will be made available by the authors, without undue reservation.

## Supplementary material

Supplementary Table 1 Irrigation volumes applied under each treatment. F (Flowing), DI (Drip Irrigation) and SI (Micro-sprinkler irrigation).

Supplementary Figure 1 Heatmap based on correlations between wheat yield components. **(A)** Heatmap based on factors that contributed to wheat yields. **(B)** Heatmap based on factors that contributed to maize yields.
