# Supplementary material for: Optimizing water and nitrogen management in a wheat–maize rotation system: synergistic increases in grain yield, resource use efficiency, and economic and environmental benefits
Source: Front Plant Sci. 2026 Feb 11;17:1769742. doi: 10.3389/fpls.2026.1769742 (PMC12932497; doi:10.3389/fpls.2026.1769742)
Supplement: Supplementary Table 1 — Irrigation volumes applied under each treatment. F (Flowing), DI (Drip Irrigation) and SI (Micro-sprinkler irrigation). [file Table1.docx]

Table S1 Irrigation volumes applied under each treatment.

| Crop/Year | Irrigation pattern | Sowing stage (kg ha^–1^) | Wintering stage (kg ha^–1^) | Elongation stage (kg ha^–1^) | Filling stage (kg ha^–1^) | Total irrigation volume (m^3^ ha^–1^) |
| --- | --- | --- | --- | --- | --- | --- |
| Wheat 2022–2023 | F | 570 | 900 | 630 | 675 | 2775 |
|  | DI | 450 | 600 | 450 | 450 | 1950 |
|  | SI | 480 | 675 | 495 | 525 | 2175 |
| Wheat 2023–2024 | F | 525 | 600 | 525 |  | 1650 |
|  | DI | 360 | 420 | 300 |  | 1080 |
|  | SI | 420 | 420 | 405 |  | 1245 |
| Maize 2023 | F | 450 |  | 450 | 675 | 1575 |
|  | DI | 345 |  | 330 | 450 | 1125 |
|  | SI | 360 |  | 375 | 570 | 1305 |
| Maize 2024 | F | 600 |  | 675 |  | 1275 |
|  | DI | 420 |  | 450 |  | 870 |
|  | SI | 450 |  | 525 |  | 975 |

Note: F (Flowing), DI (Drip Irrigation) and SI (Micro-sprinkler irrigation).
