# Supplementary figures and images for: Optimizing water and nitrogen management in a wheat–maize rotation system: synergistic increases in grain yield, resource use efficiency, and economic and environmental benefits
Source: Front Plant Sci. 2026 Feb 11;17:1769742. doi: 10.3389/fpls.2026.1769742 (PMC12932497; doi:10.3389/fpls.2026.1769742)

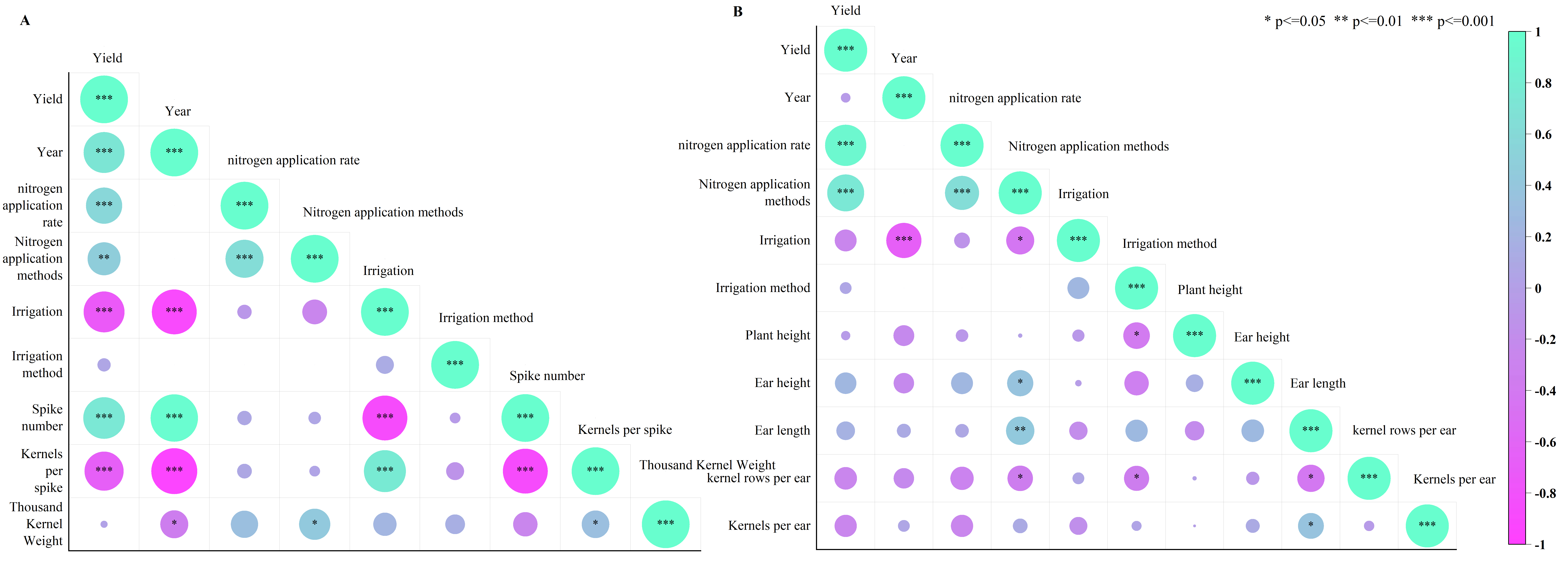

Supplement: Supplementary Figure 1 — Heatmap based on correlations between wheat yield components. (A) Heatmap based on factors that contributed to wheat yields. (B) Heatmap based on factors that contributed to maize yields. [file Image1.jpg]
